# The Cobalamin-Dependent Gene Cluster of *Listeria monocytogenes*: Implications for Virulence, Stress Response, and Food Safety

**DOI:** 10.3389/fmicb.2020.601816

**Published:** 2020-11-06

**Authors:** Justin M. Anast, Thomas A. Bobik, Stephan Schmitz-Esser

**Affiliations:** ^1^Interdepartmental Microbiology Graduate Program, Iowa State University, Ames, IA, United States; ^2^Department of Animal Science, Iowa State University, Ames, IA, United States; ^3^Roy J. Carver Department of Biochemistry, Biophysics and Molecular Biology, Iowa State University, Ames, IA, United States

**Keywords:** *Listeria monocytogenes*, food safety, virulence, stress survival, cobalamin, ethanolamine, 1, 2-propanediol, Rli47

## Abstract

Several genes of the *eut*, *pdu*, and *cob/cbi* operons are responsible for the metabolism of ethanolamine (EA) and 1,2-propanediol (PD) and are essential during the pathogenic lifecycles of various enteric pathogens. Studies concerning EA and PD metabolism have primarily focused on bacterial genera from the family *Enterobacteriaceae*, especially the genus *Salmonella*. *Listeria monocytogenes* is a member of the *Firmicutes* phylum and is the causative agent of the rare but highly fatal foodborne disease listeriosis. The *eut*, *pdu*, and *cob/cbi* operons are organized as a single large locus collectively referred to as the cobalamin-dependent gene cluster (CDGC). The CDGC is well conserved in *L. monocytogenes*; however, functional characterization of the genes in this cluster and how they may contribute to *Listeria* virulence and stress tolerance in food production environments is highly limited. Previous work suggests that the degradation pathway of PD is essential for *L. monocytogenes* establishment in the gastrointestinal tract. In contrast, EA metabolism may be more important during intracellular replication. Other studies indicate that the CDGC is utilized when *L. monocytogenes* is exposed to food and food production relevant stress conditions. Perhaps most noteworthy, *L. monocytogenes* exhibits attenuated growth at cold temperatures when a key EA utilization pathway gene was deleted. This review aims to summarize the current knowledge of these pathways in *L. monocytogenes* and their significance in virulence and stress tolerance, especially considering recent developments.

## Introduction

Foodborne pathogens must adapt to various stress conditions to survive in food production and host environments. The capacity to metabolize alternative substrates for energy is imperative for the survival of pathogens when more efficient nutrients are unavailable. Such environments include – among others – the nutrient-limited conditions within the phagosomes of macrophages. The metabolism of 1,2-propanediol (PD) and ethanolamine (EA) critically impacts the virulence of various enteric pathogens, including *Salmonella enterica*, *Enterococcus faecalis*, *Escherichia coli*, and *Listeria monocytogenes*. Enzymes required for the metabolism of PD and EA are dependent on cobalamin (vitamin B_12_) derivatives as cofactors. Cobalamin is synthesized *de novo* via the *cob* and *cbi* genes and recycled for use in these enzymatic reactions. Studies concerning the functional characterization of EA and PD metabolic genes and how they contribute to virulence have been, for the most part, conducted in *Salmonella*, *E. coli*, and *Enterococcus*. However, the ability to utilize EA and PD has been demonstrated in other distantly related genera, including *Mycobacterium*, *Corynebacterium*, *Lactococcus*, and *Listeria* as well as non-pathogenic bacteria (Nandedkar, 1974; Blackwell et al., 1976; Hartmans and Bont, 1986; Rodionov et al., 2003; Xue et al., 2008; Tsoy et al., 2009; Kutzner et al., 2016; Zeng et al., 2019).

*Listeria monocytogenes* is the causative agent of the foodborne illness listeriosis, a rare but severe disease with a high mortality rate in the immunocompromised, very young, and elderly populations. Furthermore, listeriosis also causes abortions in pregnant women (Allerberger and Wagner, 2010; Scallan et al., 2011; Radoshevich and Cossart, 2018). *L. monocytogenes* is also of considerable concern because of its long-term survival (also called persistence) in food production environments (Keto-Timonen et al., 2007; Carpentier and Cerf, 2011; Ferreira et al., 2014). *L. monocytogenes* may persist in these environments for up to decades due to increased tolerance to stresses associated with such environments (Bucur et al., 2018). Some *L. monocytogenes* strains exhibit increased tolerance toward quaternary ammonia disinfectants (Ferreira et al., 2014; Muller et al., 2014), acidic, oxidative, high or low temperatures, and high salt stress conditions (Bucur et al., 2018). Much is known concerning EA and PD metabolism genes and their contribution to *L. monocytogenes* virulence and stress conditions; however, a review that summarizes this information and highlights their comprehensive significance is absent in the scientific literature. Therefore, this review aims to summarize how EA and PD metabolism influences stress survival and virulence of *L. monocytogenes*.

## 1,2-Propanediol and Ethanolamine Metabolism

Bacterial microcompartments (BMCs) are organelle-like structures surrounded by semi-permeable protein shells encapsulating metabolic enzymes that often yield volatile toxic intermediates, thus protecting the cell from damage by mediating reactions within the BMC (Chowdhury et al., 2014; Kerfeld et al., 2018). The first BMCs, known as carboxysomes, were discovered by investigations of carbon fixation and contained high levels of ribulose-1,5-bisphosphate carboxylase oxygenase (Shively et al., 1973). Subsequent studies functionally characterized BMCs to mediate PD (Bobik et al., 1999; Sampson and Bobik, 2008) and EA (Garsin, 2010; Chowdhury et al., 2014) metabolism. Recently, Zeng et al. (2019) showed, for the first time, that BMCs form in *L. monocytogenes*, and their findings further indicated that BMCs mediate the metabolism of PD and that the addition of PD to minimal media stimulated growth in anaerobic conditions. The ability to metabolize EA in *L. monocytogenes* has been established (Kutzner et al., 2016); however, evidence of the formation of EA associated BMCs has yet to be elucidated.

PD metabolism is well documented in *Listeria.* PD, also known as propylene glycol, is a United States Food and Drug Administration approved additive to food and food containers. PD is biogenically produced from the catabolism of rhamnose or fucose and is often a fermentative end product of commensal bacteria. These sugars are highly abundant in plants, bacterial capsules, and eukaryotic glycoconjugates (Chowdhury et al., 2014). In *L. monocytogenes*, PD is metabolized by the enzymes of the *pdu* gene cluster. First, rhamnose or fucose is converted into L-lactaldehyde by aldolases that correspond to the sugar’s stereochemistry. Secondly, L-lactaldehyde is then oxidized by a propanediol oxidoreductase to form PD (Baldoma and Aguilar, 1988). The metabolism of PD produces the toxic intermediate propionaldehyde within the PD BMC, thus protecting *L. monocytogenes* from further damage. Propionaldehyde is then converted by a series of *pdu* enzymes to form propionate, propanol, or succinate (Baldoma and Aguilar, 1988; Garsin, 2010; Zeng et al., 2019).

Recent studies have revealed that *L. monocytogenes* can utilize EA as a carbon and nitrogen source. Phosphatidylethanolamine is the most abundant phospholipid in membranes of mammalian enterocytes (Kawai et al., 1974). Phosphatidylethanolamine is oxidized to glycerol and EA by non-specific phospholipases and then can freely diffuse across bacterial membranes. However, in acidic conditions, the EA transporter EutH is required for the translocation of protonated EA across cellular membranes (Anderson et al., 2018; Kaval and Garsin, 2018). *Listeria* may use EA as a carbon and nitrogen source by the further breakdown of EA to acetaldehyde and ammonia mediated by an EA ammonia-lyase known as the EutB/EutC complex (Kutzner et al., 2016; Kaval and Garsin, 2018). The EA utilization pathway genes (*eut*) can further metabolize acetaldehyde to ethanol or acetyl-CoA. Acetyl-CoA is subsequently oxidized to pyruvate or acetate to accommodate the current metabolic needs of the cell (Joseph et al., 2006; Garsin, 2010; Kaval and Garsin, 2018). For the remainder of this review, the *pdu*, *eut*, and *cbi/cob* genes will be collectively referred to as the CDGC.

## Conservation

The CDGC is highly conserved within a single large locus (Supplementary Table 1) among *Listeria sensu strictu* species representing *Listeria* species capable of growth in the gastrointestinal tracts (GI) of animals (except for the loss of one gene, *pduJ*, in *L. marthii*). However, homologs are absent in *Listeria sensu lato* species, which are exclusively environmental isolates (Buchrieser et al., 2003; Chiara et al., 2015; Schardt et al., 2017; Zeng et al., 2019), further supporting the hypothesis that PD and EA metabolism is advantageous for *Listeria* species that colonize the mammalian GI tract. It is likely that an ancestor of *Listeria sensu strictu* acquired the CDGC through horizontal gene transfer from *Bacillus cereus* (Chiara et al., 2015).

## Regulation

The regulation of PD and EA utilization has been well-characterized in *Listeria sensu strictu* species (Joseph et al., 2006; Xue et al., 2008; Mellin et al., 2013, 2014; Anderson et al., 2018; Costa and Escalante-Semerena, 2018; Zeng et al., 2019). In *L. monocytogenes*, both the *pdu* and *eut* operons require cobalamin as a cofactor for essential catabolic reactions, and PD or EA must be present for optimal expression of each respective metabolic pathway. The availability of cobalamin tightly regulates both pathways by binding to two *Cis*-acting riboswitches, Rli39 and Rli55 (Mellin et al., 2013, 2014).

The regulation of the *eut* operon in *L. monocytogenes* diverges from the well-studied pathways found in the family *Enterobacteriaceae* in that it lacks the classic EutR regulator and instead encodes a two-component response regulator (EutV/EutW) that is tightly regulated by a cobalamin-dependent riboswitch (Rli55) (Mellin et al., 2014). Many *Firmicutes*, including *L. monocytogenes*, have long *eut* operons, unlike the shorter operons found in many *Proteobacteria*, and Tsoy et al. (2009) also found that *Enterobacteriaceae* obtained the *eut* operon from *Firmicutes* and later acquired the EutR regulator by horizontal gene transfer.

The *pdu* regulon of *L. monocytogenes* shares many common traits with that of the well-characterized *pdu* regulon of *Salmonella*. PocR is the central regulator of the *pdu* genes in both organisms and is activated by PD in the absence of more efficient carbon sources. However, the *pdu* operon of *Salmonella* is controlled differently on a global scale through the mediation of a cyclic AMP receptor protein-cyclic AMP complex and the ArcA/ArcB system (Chowdhury et al., 2014). In *L. monocytogenes*, *pocR* gene expression is finely tuned by a cobalamin-dependent riboswitch (Rli39) that, in the absence of cobalamin, will generate a longer transcript that is antisense of *pocR* (as*pocR*), thereby hindering the translation of *pocR* mRNA. After translation, PocR is activated in the presence of PD. PocR acts as a positive regulator of the *pocR* gene (ensuring its production while PD is present) and will, in turn, activate the *pdu* and possibly the *cob* and *cbi* genes, thereby helping maintain production of cobalamin derived cofactors and the utilization of PD (Mellin et al., 2013). Several *L. monocytogenes pdu* and *cob* genes are regulated on a global scale directly by the stress regulator σ^B^ (Liu et al., 2017), and are directly or indirectly influenced by the non-coding RNA, Rli47 (Marinho et al., 2019). Rli47 has recently emerged as an important regulatory element in virulence and especially during stress response (Toledo-Arana et al., 2009; Archambaud et al., 2012; Mujahid et al., 2012; Marinho et al., 2019; Anast and Schmitz-Esser, 2020; Cortes et al., 2020).

## Virulence

The importance of the CDGC in the pathogenicity of many enteric pathogens such as *Salmonella*, *Escherichia*, *Enterococcus*, and *Clostridium*, is well established (Garsin, 2010; Thiennimitr et al., 2011; Nuccio and Baumler, 2014; Faber et al., 2017). In *Salmonella* and *Escherichia*, the *eut* gene regulator EutR is essential for the development of disease because, in addition to regulating the *eut* genes, EutR also influences key virulence operons. Known EutR-dependent virulence factors are *Salmonella* genes of the pathogenicity island 2 (SPI-2), including the SPI-2 positive gene regulator, *ssrB* (Anderson et al., 2015); as well as *E. coli* virulence genes *ler*, *qseC*, *stx2a*, and an important type III secretion system (LEE effacement locus) (Kendall et al., 2012; Luzader et al., 2013; Gonyar and Kendall, 2014). During intestinal inflammation caused by *Salmonella* infection, it was found that microbiota-derived EA and PD were utilized as electron donors in respiration with tetrathionate as the required final electron acceptor (Thiennimitr et al., 2011; Faber et al., 2017). Interestingly, respiration with electron donors EA and PD promoted *Salmonella* expansion in the presence of commensal bacteria, but not during infection of germ-free mice. These data suggest that the utilization of EA and PD enhances *Salmonella* virulence in the GI tract by shifting metabolism towards respiration on commensal microbiota fermentative byproducts and electron acceptors made available through intestinal inflammation. Also, *Salmonella* (Nuccio and Baumler, 2014; Matthews et al., 2015) and commensal *E. coli* (Dogan et al., 2014) isolates not associated with GI disease often have absent or disrupted PD utilization operons, and it was found that bacteria that are associated with food poisoning more often possessed the long-form *eut* operon containing BMC shell component genes (like that of *L. monocytogenes*) (Tsoy et al., 2009).

The CDGC has recently been highlighted to be important for *L. monocytogenes* pathogenicity. A study that used a tiling array approach showed that the *pdu* and *eut* genes were significantly upregulated by *L. monocytogenes* in the GI tract and blood of mice (Toledo-Arana et al., 2009). Interestingly, a deletion mutant of the *L. monocytogenes pduD* gene showed attenuated virulence and faster clearing of *L. monocytogenes* in the GI tract, highlighting the importance of PD utilization for the fitness of *L. monocytogenes* during infection in the GI tract (Schardt et al., 2017). Archambaud et al. (2012) found that the expression of the CDGC was induced in the GI tract of gnotobiotic mice during orally acquired listeriosis compared to growth in broth. Remarkably, they found a much higher expression of these genes in the transcriptomic response of *L. monocytogenes* when *Lactobacillus* was present in the GI tract (Archambaud et al., 2012).

These data suggest that the utilization of the CDGC in the GI tract may increase competitive fitness over commensal bacteria (that can only metabolize fermentable carbon sources) by enabling *L. monocytogenes* to grow on non-fermentable carbon and nitrogen sources. Enhancing *L. monocytogenes* competitive fitness in the GI tract may increase the likelihood of successful invasion into the intestinal epithelium.

Once *L. monocytogenes* establishes in the GI tract, it internalizes into the epithelial cells (EC) of the host by hijacking host cellular machinery, and it then proceeds to replicate intracellularly (Radoshevich and Cossart, 2018). Additionally, *L. monocytogenes* can translocate the epithelial lining via the paracellular route after the partitioning of host cell tight junctions, mediated by the *Listeria* adhesion protein LAP (Drolia and Bhunia, 2019). Both *pdu* and *eut* genes were significantly induced during replication in caco-2 cells (a human EC line); however, only an insertion knockout mutant of *eutB*, which mediates the first step in EA metabolism, exhibited a decrease in intracellular replication. Δ*eutB* displayed significant attenuation of intracellular growth, second only to mutants of *inlA*, the gene encoding the protein responsible for internalization into ECs (Joseph et al., 2006). It has been proposed that non-specific phospholipases, such as PlcA/PlcB, cleave phosphatidylethanolamine from host cell membranes for utilization by intracellularly replicating *L. monocytogenes* (Joseph et al., 2006; Mellin et al., 2014; Staib and Fuchs, 2014).

The next crucial step in the systemic spread of *L. monocytogenes* is disseminating to peripheral organs via uptake and replication in monocytes, such as macrophages (Radoshevich and Cossart, 2018). Mellin et al. (2014) found that an *eutB* deletion mutant was highly attenuated in an intravenous mouse infection model and exhibited significantly lower numbers of *L. monocytogenes* in spleen and liver cells. Macrophages are phagocytic cells and will autonomously engulf invading bacteria within vacuoles known as phagosomes. Bacteria-containing phagosomes will then combine with lysosomes containing toxic substances such as reactive oxygen species. The fusion will form a phagolysosome that will destroy the confined bacteria. For *L. monocytogenes* to survive transient or extended periods within these vacuoles, it must employ a repertoire of virulence factors. The environment within these macrophagic vacuoles is acidic, and it is known that *L. monocytogenes* excretes Listeriolysin O (LLO) and the phospholipases PlcA/PlcB to promote neutralization and escape from host cell vacuoles (Radoshevich and Cossart, 2018). EA can freely permeate bacterial cell membranes at neutral pH. However, EA is protonated in acid conditions and requires the EA permease, EutH, to be internalized by *L. monocytogenes*. Anderson et al. (2018) elucidated that EutH is important for the intracellular survival and replication of *S. Typhimurium* and *L. monocytogenes*. EutH did not promote intestinal infection but instead provides a crucial advantage in the dissemination of *L. monocytogenes* to the liver and spleen. At 2 h post-infection of macrophages by *L. monocytogenes*, a deletion mutant of *eutH* (Δ*eutH*) exhibited a similar reduction in cell numbers as a mutant of the gene encoding LLO when compared with the wildtype control. The Δ*eutH* detrimental phenotype was not observed at 5 or 8 h post internalization; however, the LLO mutant consistently showed a high reduction of intracellular derived CFUs. This may be explained by the fact that LLO has essential functions in many aspects of intramacrophage survival (Osborne and Brumell, 2017) and that once *L. monocytogenes* is free of the acidic conditions of the phagosome, EutH does not have an essential role in survival (Osborne and Brumell, 2017; Anderson et al., 2018). *L. monocytogenes* has been shown to escape from phagosomes as early as 30 min post macrophage internalization (Tilney and Portnoy, 1989; Myers et al., 2003). However, the majority of *L. monocytogenes* cells remains confined to the phagosome 2–3 h post-infection (de Chastellier and Berche, 1994; Singh et al., 2008). Anderson et al. (2018) also proposed that there may be a functional link between LLO and EutH; however, a hypothesis that suggests a function was not proposed in the study. These findings highlight the importance of EutH for the survival and replication of *L. monocytogenes* in macrophages in response to vacuole acidification.

Interestingly, the first step in EA metabolism is the oxidation of EA to acetaldehyde and ammonia (NH_3_) by the EutB/EutC complex. In S*almonella*, it is suggested that the EutB/EutC complex is associated with the outer surface of the BMC and injects the EutB/EutC-derived acetaldehyde into the lumen of the BMC shell (Chowdhury et al., 2014). Ammonia likely remains in the cytosol, where it can be incorporated into nitrogen metabolism. Phagolysosomes of host cells can indeed mediate nutrient capture and sequester metals such as Fe^2+^ and Zn^2+^ (Uribe-Querol and Rosales, 2017), and EA may serve as the sole nitrogen and carbon source within these vacuoles. *L. monocytogenes* and other bacteria, such as *Salmonella*, may synthesize and recycle cobalamin via ATP:Co(I)rrinoid adenosyl transferases denoted for their respective pathway: CobA (for the *de novo* synthesis of cobalamin), EutT, or PduO (Chowdhury et al., 2014; Costa and Escalante-Semerena, 2018; Zeng et al., 2019). Until recently, ATP:Co(I)rrinoid adenosyl transferases were described as requiring metal ions such as Fe(II) or Zn(II) for optimal activity; however, Costa and Escalante-Semerena (2018) have described a new class of EutT in *L. monocytogenes* which does not require metal ions for activity.

Therefore, it seems likely that EA metabolism may benefit *L. monocytogenes* during nutrient-limited intra-vacuolar replication. Interestingly, the impairment of protonated-EA import reduced intramacrophage cell survivability after 2 h, similar to an LLO mutant (Anderson et al., 2018). If EA metabolism only increased fitness through nutrient acquisition, it would be suspected that intracellular replication may be hindered. However, Δ*eutH* CFUs (determined after intramacrophage growth) were reduced by 42% when comparing 0–2 h post-infection (Anderson et al., 2018), suggesting that cell death occurred. Is it possible that the increased production of ammonia during the first step of EA metabolism may neutralize free protons that can freely permeate the cell membrane? Is it also possible that the secretion of ammonia provides localized protection against the acidic conditions of the phagosome? LLO forms pores that increase in size over time, destabilizing the pH and membrane integrity of the phagosomes. Could the time-dependent effectivity of LLO pore formation in the phagosome membrane explain why Δ*eutH* only exhibits a reduction of cell survivability and growth at 2 h post macrophage phagocytosis (Anderson et al., 2018)? The *L. monocytogenes* genome encodes a transporter that is predicted to be involved with ammonia/ammonium transport (*lmo1516*), and it has been shown that ammonia transport is passive and bidirectional in other bacteria (Wingreen, 2004).

**FIGURE 1:**
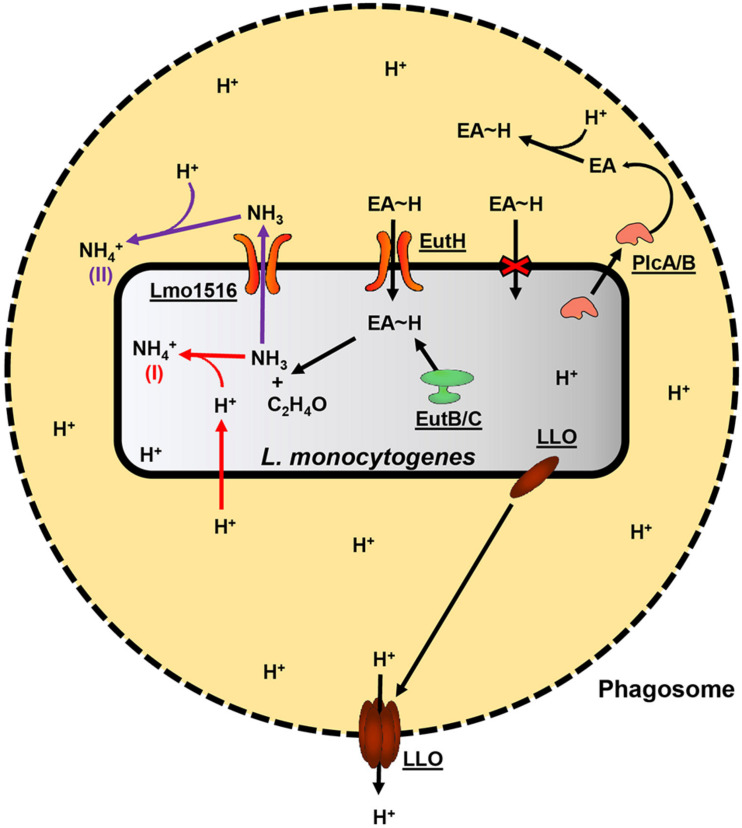
Proposed model of EA oxidation and its contribution to the pathogenicity of *L. monocytogenes.* A black dashed perimeter indicates the membrane of the acidic phagosome. Free protons are denoted “H^+^,” and protein names or *L. monocytogenes* EGD-e locus tags are underlined. *L. monocytogenes* secretes LLO and PlcA/PlcB during its lifecycle within host-cell phagosomes. LLO will associate with the membrane of the phagosome and form pores, allowing protons to diffuse into the host cell cytosol, which, in turn, raises the internal pH of the phagosome. PlcA/PlcB may assist vesicle escape by degrading the host phospholipid membranes. In addition to the already present EA, degrading phosphatidylethanolamine found in host membranes would release additional EA into the vesicle environment. EA is quickly protonated (EA∼H) in the acidic conditions of the phagosome and is internalized by *L. monocytogenes* by the EutH transporter. The oxidation of the imported EA by the EutB/EutC complex yields acetaldehyde and ammonia. The acetaldehyde and ammonia may enter general carbon and nitrogen metabolism; however, the production of ammonia may contribute to the survival of *L. monocytogenes* during intracellular life in at least two mechanisms. In the first mechanism, denoted by a red “(I)” and red arrows, ammonia spontaneously reacts with a free proton (which may freely diffuse across *L. monocytogenes* cell membrane) within the *Listeria* cytosol and thereby increase intracellular pH by producing ammonium. In the second mechanism, denoted by a purple “(II)” and purple arrows, ammonia is excreted from *L. monocytogenes* and spontaneously reacts with protons outside the *Listeria* cell, yielding ammonium, and thereby providing a degree of localized protection from the acidic conditions of the phagosome.

*Mycobacterium tuberculosis*, *Helicobacter pylori*, and *Staphylococcus aureus* utilize ammonia production for localized protection against and possibly to inhibit host defenses such as the formation of phagolysosomes and acidic conditions (Gordon et al., 1980; Ansari and Yamaoka, 2017; Zhou et al., 2019). PlcA/PlcB are supportive in the escape from host phagosomes, possibly by the degradation of the phagosome phospholipid membrane. However, PlcA/PlcB are not required for intracellular growth, unlike LLO, and the exact mechanism of how PlcA/PlcB supports escape from the phagosome is unknown (Lam et al., 2012; Radoshevich and Cossart, 2018). Therefore, we hypothesize that ammonia produced in the initial step of EA catabolism has an essential role during *L. monocytogenes* pathogenesis. Ammonia derived from EA metabolism could provide localized protection from the acidic conditions within the macrophage phagosome by neutralizing low pH. We further hypothesize that PlcA/PlcB contributes to the survival within macrophage phagosomes by degrading phagosome membranes. The degradation of the phagosome membrane releases EA from phosphatidylethanolamine, which *L. monocytogenes* can metabolize and convert to ammonia. A model of how *L. monocytogenes* may use EA metabolism during virulence is proposed in Figure 1. This hypothesis could be tested in future experiments by generating deletion mutants of the *lmo1516* putative ammonia/ammonium transporter gene and comparing the deletion mutant with the wildtype to identify possible effects on the survival in macrophages.

Thus, the analysis of the CDGC in *L. monocytogenes* seems to indicate that both EA and PD utilization is crucial in the establishment of *L. monocytogenes* in the GI tract, and the presence of *eut* genes and EA utilization may promote the systemic spread and intracellular replication during listeriosis infections. Additionally, EA may serve as a nutrient source within the macrophage cytosol. Once *L. monocytogenes* escapes from the macrophage phagosome, EA is available in phospholipids derived from the destroyed phagosome membrane. However, it seems more likely that, since EA metabolism is not as energetically favorable as the utilization of other nitrogen and carbon sources, and *L. monocytogenes* likely shifts metabolism toward more easily metabolizable nutrient sources (Sauer et al., 2019). A possible utilization of EA by *L. monocytogenes* after macrophage escape would need to be verified in future experiments.

## Implications for Survival in Food and Food Production Environments

Although it is well established that the CDGC may contribute to the survival of foodborne pathogens in food and food production environments (Chan et al., 2008; Fox et al., 2011; Srikumar and Fuchs, 2011; Goudeau et al., 2013; Tang et al., 2015; Kaval and Garsin, 2018; Anast and Schmitz-Esser, 2020), functional characterizations of the *L. monocytogenes* CDGC importance during food and food production associated stresses are far more limited than studies that assess its contribution to virulence. PD and EA are found in many food products as constituents of glycoconjugates or lipids and are used as additives of various food products and containers (Cameron et al., 1998; Tang et al., 2015). Tang et al. (2015) found that *L. monocytogenes* grown on cold vacuum-packed salmon significantly increased expression of the CDGC compared to growth in rich media. They proposed that *L. monocytogenes* induced the CDGC to utilize PD and EA found in salmon, increasing proliferation on the food matrix within the vacuum packaging (Tang et al., 2015). *Carnobacterium piscicola* is naturally found on salmon and can inhibit the growth of *Listeria*. When *C. piscicola* was co-cultured with *L. monocytogenes*, it was elucidated that *C. piscicola* attenuated the growth of *L. monocytogenes* partially by glucose depletion. During this inhibition, *L. monocytogenes* significantly increased the expression of the CDGC (Nilsson et al., 2005). *L. monocytogenes* also increased expression of the *pdu* gene cluster in co-culture biofilm with *Bacillus subtilis* but not in mono-culture biofilm (Tirumalai, 2015). Recently, a study from our laboratory that co-cultured *L. monocytogenes* with either a *Brevibacterium* or *Psychrobacter* food isolate found that the CDGC was highly upregulated in a modular way and was dependent on co-culture condition (Anast and Schmitz-Esser, 2020). We also observed that Rli47 was by far the most expressed gene during co-cultivation, further linking the functions of Rli47 and the CDGC (Anast and Schmitz-Esser, 2020). The authors of some recent studies (Tang et al., 2015; Tirumalai, 2015; Anast and Schmitz-Esser, 2020) concur that *L. monocytogenes* is most likely searching for alternative nutrients to fit into competitive niches, perhaps in a more cooperative approach.

Certain strains of *L. monocytogenes* are primarily known for their prolonged persistence in food production environments (Carpentier and Cerf, 2011; Ferreira et al., 2014). These strains often harbor genes that confer increased tolerance to stresses associated with these habitats, i.e., protection against methods used to control microbial growth in food production operations. The literature strongly suggests that the CDGC may provide survival advantages to *L. monocytogenes* during exposure to disinfectants, growth inhibitors, desiccation, and during cold temperature (Chan et al., 2008; Fox et al., 2011; Mattila et al., 2012; Tessema et al., 2012; Hingston et al., 2017; Suo et al., 2018; Kragh and Truelstrup Hansen, 2019; Cortes et al., 2020).

In one study, the transcriptional profiles of a persistent vs. a non-persistent *L. monocytogenes* strain were assessed in response to exposure to the quaternary ammonia compound disinfectant, benzethonium chloride (Fox et al., 2011). In the persistent strains, the expression of the CDGC was highly upregulated compared to the non-persistent strains. This study concluded that the utilization of this operon in response to disinfectant stress might confer a survival advantage to *L. monocytogenes*, therefore prolonging persistence in food production facilities (Fox et al., 2011). Many growth inhibitors, such as organic acids, are commonly added to foods to inhibit the growth of *Listeria* (Bucur et al., 2018). The *eut* two-component response regulator EutV/EutW, the EA transporter EutH and the positive regulator of PD metabolism PocR were all downregulated when *L. monocytogenes* was exposed to media acidified to pH five by hydrochloric, acetic, or lactic acid (Tessema et al., 2012). In contrast, another study found that *pocR* and *eutV/eutW* were highly induced during lactic acid exposure at pH 3.4, suggesting that the induction of the CDGC in response to acidic stress may be highly dependent on condition (Cortes et al., 2020). Sodium lactate is a widely used food additive that does not influence pH, enhances the organoleptic properties of meat products, and may increase the water compacity of foods (Papadopoulos et al., 1991). When *L. monocytogenes* was exposed to 4% (wt/wt) sodium lactate and cultivated in ready-to-eat meat products, nearly the entire CDGC (*n* = 66 genes) was highly downregulated (Suo et al., 2018).

Limiting or removing water from food and food production environments is a standard method of microbial control. When *L. monocytogenes* was desiccated for short and extended timepoints on steel plates, it was revealed that 16 CDGC genes altered in expression (11 upregulated and five downregulated). Interestingly, both *eutV/eutW* were downregulated in all five measured timepoints (Kragh and Truelstrup Hansen, 2019). Maintaining food at cold temperatures is another prominent method used for controlling the growth of pathogens and other spoilage organisms. The transcriptional response of *L. monocytogenes* was also assessed regarding different temperatures relevant to the food production environment (20 vs. 4°C). Remarkably, the authors of a recent study found that at 4°C *L. monocytogenes* increased expression of 17 genes of the *eut* pathway, while in contrast, many of the *pdu* genes and almost the entire operon for *de novo* synthesis of cobalamin was downregulated (Hingston et al., 2017). This study also observed high expression levels of as*pocR*, suggesting that PocR did not induce the *pdu* genes. The consistent induction of the *eut* genes and repression of the *pdu* genes at colder temperatures may indicate that EA may be more preferably metabolized at low temperatures than PD. This theory is supported by a previously unrecognized precedent of the importance of EA metabolism during cold temperatures in *L. monocytogenes*. Chan et al. (2008) created deletion mutants of *L. monocytogenes* sigma factors, two-component regulatory systems, and negative regulators to investigate their contribution during cold adaptation. Interestingly, the response regulator *eutV* deletion mutant had significantly lower cell counts than the wildtype after 12 days of incubation at 4°C (Chan et al., 2008). Later, a homolog of *eutV* in *Enterococcus faecalis* was described to be a part of the two-component response regulatory system controlling the EA metabolism genes (Del Papa and Perego, 2008; Fox et al., 2009). Noteworthy, the genomic organization of the *eut* gene pathway is conserved between *Enterococcus faecalis* and *L. monocytogenes*. Lastly, Mattila et al. (2012) demonstrated that during exposure of *L. monocytogenes* to 3 and 37°C, both *eutV/eutW* are positively regulated by σ^L^, the alternative sigma factor shown to be required for efficient growth during low temperatures and organic acid exposure. Remarkably, PocR was positively influenced by σ^L^ during growth at 3°C but in contrast, was downregulated by σ^L^ during cultivation at 37°C. These transcriptomic data suggest that σ^L^ positively regulates both *pdu* and *eut* genes at colder temperatures, but represses PocR at optimal temperature.

The studies mentioned above suggest that the CDGC, especially the EA utilization module, contributes to the survival of *L. monocytogenes* in food and food production environments. The deletion of one *eut* gene leads to impaired growth at refrigeration temperatures. However, the mechanism of how EA metabolism may be necessary for the proliferation of *L. monocytogenes* at cold temperatures is currently unknown. It is tempting to speculate that PD and EA may be more readily utilized during exposure to food production environment-associated stress conditions than other, more efficient carbon and energy substrates such as glucose.

## Conclusion and Future Directions

Although evident attenuation of virulence is observed when EA and PD metabolism genes are deleted, the molecular mechanisms of how key EA and PD metabolic genes contribute to pathogenicity remain unknown in *Listeria*. Although several transcriptomic studies show that the CDGC is differentially expressed during food and food production environment relevant stress conditions, functional characterization of how CDGC mechanisms may contribute to *L. monocytogenes* stress tolerance is, to the best of our knowledge, currently unavailable. Thus, determining the contribution of the CDGC to survival under food and food production associated stress conditions may – in the future – reveal novel countermeasures for implementation into good management practices of food production plants. Finally, at least two alternative sigma factors, a two-component regulatory system, non-coding RNAs such as Rli39, as*pocR*, Rli47, and Rli55, are involved in regulating the CDGC, emphasizing the complexity of the regulatory network of CDGC. Alternative sigma factors and non-coding RNAs have emerged as critical regulatory entities in both virulence and general stress response that may have global impacts on gene expression.

One important aspect of CDGC that should be addressed in future research is to identify whether EA catabolism may have an effect on the host immune system and commensal bacteria of the intestine. Acetate is the final end-product of EA catabolism, and it has been shown to modulate the immune response by suppressing IgA production in the GI tract (Maslowski et al., 2009; Wu et al., 2017). It is possible that EA metabolism resulting in the production of acetate by enteric pathogens disrupts the host immune response. This hypothesis has been previously proposed by others (Kaval and Garsin, 2018), and to the best of our knowledge, has yet to be experimentally verified. In addition, it may be important to test if the utilization of PD is restricted to the intestinal mucosa as a source of rhamnose or fucose. These two sugars are released from complex carbohydrates by commensal bacteria in the GI tract and may not be available in the host blood, lymphatic fluid, or during survival in monocytes and hepatocytes in concentrations or forms that are feasible for *L. monocytogenes* to metabolize them. Possible ways to test if the catabolism of these sugars is essential for the intestinal survival of *L. monocytogenes* would be to delete key genes involved in the metabolism of fucose, rhamnose, and PD and then test *L. monocytogenes* survival or growth in the GI tract, or during *in vitro* growth by using defined media with and without fucose and rhamnose.

In future studies that assess the CDGC of *L. monocytogenes*, experiments should be designed to observe the global effects of EA and PD metabolism by transcriptomic and non-targeted metabolomic approaches to best represent the comprehensive phenotype of *L. monocytogenes* in regards to the tested condition. Much of the CDGC of *L. monocytogenes* and its contribution to virulence and stress survival has been elucidated, but further study should be encouraged to uncover more specific mechanisms in which the CDGC enables *Listeria* to survive food and food production associated stress conditions, which can subsequently lead to foodborne illness.

## Author Contributions

JA conceived and wrote the first draft of the manuscript and analyzed data. SS-E, TB, and JA wrote and approved the final manuscript. All authors contributed to the article and approved the submitted version.

## Conflict of Interest

The authors declare that the research was conducted in the absence of any commercial or financial relationships that could be construed as a potential conflict of interest.
