# Supplementary material for: The Cobalamin-Dependent Gene Cluster of Listeria monocytogenes: Implications for Virulence, Stress Response, and Food Safety
Source: Front Microbiol. 2020 Nov 6;11:601816. doi: 10.3389/fmicb.2020.601816 (PMC7677406; doi:10.3389/fmicb.2020.601816)
Supplement: Supplementary file 1 [file Data_Sheet_1.PDF]

Table S1. The structure and predicted regulatory elements of the *Listeria monocytogenes* cobalamin-dependent gene cluster

EGD-e locus tag	Gene name	Product	Regulation
lmo1141	*cobU*	Siroheme synthase subunit family protein	
lmo1142	*pduS*	conserved protein of unknown function	PocR
lmo1143	*pduT*	PduT	
lmo1144	*pduU*	Putative carboxysome-like ethanolaminosome structural protein with a putative role in ethanol utilization	PocR
lmo1145	*pduV*	Propanediol utilization protein PduV	
lmo1146	non-*pdu* gene	Conserved protein of unknown function	
lmo1147	*cobU/copB*	Bifunctional cobinamide kinase and cobinamide phosphate guanylyltransferase	Rli47
lmo1148	*cobS*	Cobalamin synthase	
lmo1149	non-*pdu* gene/*cob* gene	Alpha-ribazole phosphatase	σ^B
lmos_41	Rli39/as*pocR*	Rli39 cobalamin riboswitch - regulates *pocR* gene expression	cobalamin
lmo1150	*pocR*	Regulatory protein PocR	PD, PocR, Rli39-as*pocR*, σ^L
lmo1151	*pduA*	Putative carboxysome-like ethanolaminosome structural protein, ethanolamine utilization protein	PocR/σ^B
lmo1152	*pduB*	Propanediol utilization protein PduB	PocR/σ^B
lmo1153	*pduC*	Propanediol dehydratase large subunit	PocR/σ^B
lmo1154	*pduD*	Propanediol dehydratase medium subunit	PocR/σ^B
lmo1155	*pduE*	Propanediol dehydratase small subunit	PocR
lmo1156	*pduG*	Diol dehydratase-reactivating factor alpha subunit	σ^B
lmo1157	*pduH*	Conserved protein of unknown function	Rli47
lmo1158	*pduK*	Conserved protein of unknown function	
lmo1159	*pduJ*	Putative carboxysome-like ethanolaminosome structural protein, ethanolamine utilization protein	σ^B
lmo1160	*pduL*	Phosphate propanoyltransferase	
lmo1161	*eutJ*	Ethanolamine utilization protein EutJ	Rli47

lmo1162	*pduM*	Conserved protein of unknown function	
lmo1163	*pduN*	Carbon dioxide concentrating mechanism protein CcmL	
lmo1164	*pduO*	ATP:cob(I)alamin adenosyltransferase protein PduO	σ^B
lmo1165	*pduP*	Putative aldehyde dehydrogenase, ethanolamine utilization protein	
lmo1166	*pduQ*	Propanol dehydrogenase	
lmo1167	*pduF*	Glycerol permease	
lmo1168	*pduW*	Acetate kinase	σ^B
lmo1169	*cobD*	Threonine-phosphate decarboxylase	
lmo1170	*pduX*	Propanediol utilization protein PduX / L-threonine kinase in cobalamin biosynthesis	Rli47
lmos_43	*rli55*	Rli55 cobalamin riboswitch - regulates *eut* gene expression - constitutive promoter	B_{12}
lmo1171	*eutG*	Alcohol dehydrogenase	EutV
lmo1172	*eutV*	Response regulator to the sensor kinase (EutW)	Rli55/EutW/EutV/σ^L
lmo1173	*eutW*	Ethanolamine sensory transduction histidine kinase	EA/EutV/σ^L
lmo1174	*eutA*	Ethanolamine utilization protein EutA	EutV
lmo1175	*eutB*	Ethanolamine ammonia-lyase, large subunit, heavy chain	
lmo1176	*eutC*	Ethanolamine ammonia-lyase, small subunit (light chain)	
lmo1177	*eutL*	Putative carboxysome-related structural protein with a putative role in ethanolamine utilization	
lmo1178	*eutK*	Conserved protein of unknown function	
lmo1179	*eutE*	Aldehyde dehydrogenase	
lmo1180	*eutM*	Putative carboxysome-like ethanolaminosome structural protein, ethanolamine utilization protein	
lmo1181	*eutT*	ATP:cob(I)alamin adenosyltransferase protein - non-medal binding requirement	
lmo1182	*eutD*	Phosphate propanoyltransferase	
lmo1183		Conserved protein of unknown function	
lmo1184	*eutN*	Carbon dioxide concentrating mechanism protein CcmL	
lmo1185		Conserved protein of unknown function	
lmo1186	*eutH*	Ethanolamine transporter EutH	

lmo1187	*eutQ*	Conserved protein of unknown function	
lmo1188		Conserved protein of unknown function	
lmo1189		Conserved protein of unknown function	
lmo1190	*cblT*	Putative B_{12} precursor transporter – PocR binding site upstream	Rli-undefined
undefined	*rli57*	Rli57 putative cobalamin riboswitch - repression of *cbiA* and possibly *cblT*	cobalamin
lmo1191	*cobB/cbiA*	Cobyrinic acid A, C-diamide synthase	Rli-undefined
lmo1192	*cobD*	Cobalamin biosynthesis protein CobD	
lmo1193	*cbiC*	Cobalt-precorrin-8X methylmutase	
lmo1194	*cbiD*	Putative cobalt-precorrin-6A synthase [deacetylating]	Rli47
lmo1195	*cbiE*	Putative cobalt-precorrin-6Y C(5)-methyltransferase	
lmo1196	*cbiT*	Putative cobalt-precorrin-6Y C(15)-methyltransferase [decarboxylating]	
lmo1197	*cbiF*	Cobalt-precorrin-4 C(11)-methyltransferase	
lmo1198	*cbiG*	CbiG protein	
lmo1199	*cbiH*	Cobalt-precorrin-3B C(17)-methyltransferase	
lmo1200	*cbiJ*	Precorrin-6X reductase	
lmo1201	*hemD*	Uroporphyrin-III C-methyltransferase/uroporphyrinogen-III synthase	Rli47
lmo1202	*cbiK*	Sirohydrochlorin cobaltochelatase	
lmo1203	*cibL*	Precorrin-2 C20-methyltransferase	
lmo1204	*cbiM*	Cobalt transport protein CbiM	
lmo1205	*cbiN*	Cobalt transport protein CbiN	
lmo1206	*cbiQ*	Cobalt transport protein CbiQ	
lmo1207	*cbiO*	Putative enzyme	
lmo1208	*cobP*	Cobyric acid synthase	
lmo1209	*pduO/cobA*	Putative ATP:cob(I)alamin adenosyltransferase	
